# Quorum Sensing and the Use of Quorum Quenchers as Natural Biocides to Inhibit Sulfate-Reducing Bacteria

**DOI:** 10.3390/antibiotics5040039

**Published:** 2016-12-15

**Authors:** Giantommaso Scarascia, Tiannyu Wang, Pei-Ying Hong

**Affiliations:** King Abdullah University of Science and Technology (KAUST), Water Desalination and Reuse Center (WDRC), Biological and Environmental Sciences and Engineering Division (BESE), Thuwal 23955-6900, Saudi Arabia; giantommaso.scarascia@kaust.edu.sa (G.S.); tiannyu.wang@kaust.edu.sa (T.W.)

**Keywords:** dissimilatory sulfate reduction, extremophiles, biofilm, biofouling, biocorrosion

## Abstract

Sulfate-reducing bacteria (SRB) are one of the main protagonist groups of biocorrosion in the seawater environment. Given their principal role in biocorrosion, it remains a crucial task to develop strategies to reduce the abundance of SRBs. Conventional approaches include the use of biocides and antibiotics, which can impose health, safety, and environmental concerns. This review examines an alternative approach to this problem. This is achieved by reviewing the role of quorum sensing (QS) in SRB populations and its impact on the biofilm formation process. Genome databases of SRBs are mined to look for putative QS systems and homologous protein sequences representative of autoinducer receptors or synthases. Subsequently, this review puts forward the potential use of quorum quenchers as natural biocides against SRBs and outlines the potential strategies for the implementation of this approach.

## 1. Introduction

The increasing scarcity of freshwater has resulted in the increasing use of seawater as a source for various industrial water applications. Examples of such usage include seawater cooling tower systems and seawater injection for enhanced oil recovery. Across all industrial sectors, metal pipelines are commonly used because of their ductility, strength, and high heat conductivity. However, metallic pipelines are prone to corrosion. This is especially exacerbated by the use of seawater as the high chloride, sulfate, and oxygen content in seawater can speed up corrosion rates through scaling and microbial induced corrosion (MIC) [1,2]. Internationally, one ton of steel turns into rust every 90 s [3] and the associated costs of metal corrosion have been estimated to range between 2% and 3% of the gross domestic product (GDP) of developed countries like the US [4].

MIC is believed to account for 20% of the damage caused by corrosion [5]. Although scaling plays a comparatively larger role in corrosion, it is relatively more well-understood compared to MIC, which was only recognized to increase corrosion rates by several orders of magnitude a few decades ago [6]. Approaches to mitigate the role of scaling in corrosion have been put in place (e.g., utilizing a once-through seawater cooling tower, the addition of water softeners to control alkalinity, and acid treatment). Another commonly used strategy to tackle corrosion in seawater pipelines includes the deaeration of seawater. In aerated seawater, oxygen concentration gradients on the pipeline surface can cause pitting and/or crevice corrosion at localized sites. Once pitting is initiated, the propagation rate is accelerated with increasing dissolved oxygen (DO) content [7]. Deaeration is thus performed to remove DO from the seawater. Deaeration can be accomplished by chemical treatment, vacuum deaeration, or countercurrent gas stripping. Vacuum deaeration and countercurrent gas stripping are commonly followed by oxygen scavenging by reacting residual oxygen with sulfite ions in the presence of a suitable catalyst.

However, deaeration and oxygen scavenging of injection water with sulfite ions creates anaerobic environments in the water, favoring the proliferation of sulfate-reducing bacteria (SRB) and subsequent SRB-mediated MIC. SRB damage metal pipelines through a corrosive chemical agent known as hydrogen sulfide, which is formed during biological sulfate reduction (i.e., chemical microbially influenced corrosion; CMIC). On the other hand, certain SRB can also attack metal pipelines via the direct extraction of electrons (i.e., electrical microbially influenced corrosion; EMIC) [6].

The conventional approach to tackling MIC is to dose biocides in large quantities (such as chlorine, ozone, and commercially available biocides that are either formulated based on formaldehyde, glutaraldehyde, or quaternary ammonium) [8]. The use of these highly toxic biocides has resulted in the consideration of their potential health, safety, and environmental impacts. According to OSHA, all materials capable of releasing formaldehyde at levels above 0.5 mg/L should be classified as potential cancer hazards [9]. The use of seawater cooling towers and the development of oil reservoirs in environmentally sensitive areas, coupled with stricter environmental regulations, has spurred interest in the use of “green biocides” that are less toxic.

In this review, we aim to present quorum sensing inhibition as a possible natural biocidal approach to tackling the main protagonist of MIC—sulfate-reducing bacteria (SRB). The review begins by providing a basic understanding of the problems caused by SRB and the genetic traits behind sulfate respiration. Secondly, a description of the current methods used to tackle SRB and microorganisms in general is presented. Finally, this review introduces the use of quorum quenching as an alternative method to tackle SRB, particularly those that cause biocorrosion problems in extreme environments such as seawater injection systems and seawater cooling towers.

### 2.1. Sulfate Reducers: Phylogenetic Affiliation and Their Contributory Roles in Industrial Problems

Sulfate reducers are ubiquitous anaerobic microorganisms that use sulfate as an electron acceptor to oxidize hydrogen and organic compounds (e.g., lactate, acetate, malate, butyrate) [10] (Table 1). They are also able to reduce sulfite, thiosulfate, and sulfur to hydrogen sulfide (H_2_S). The detection of sulfate reducers based on 16S rRNA gene sequencing analysis suggests that they can be found in both Bacteria and Archaea domains, although the bacterial SRB are more commonly found in most studied ecosystems. Within bacterial SRB, the most abundant group is composed of gram-negative Deltaproteobacteria. Clostridia is the second most abundant group, with only three gram-positive genera that include *Desulfosporosinus*, *Desulfotomaculum*, and *Desulfosporomusa*. The rest of the sulfate-reducing bacteria belong to *Nitrospirae*, the thermophile *Thermodesulfobacteria*, and *Thermodesulfobium*. Additionally, *Archaeoglobus, Themocladium*, and *Caldivirga* are three sulfate-reducing genera in the Archaea domain that have been found in extreme environment such as hot springs and hydrothermal vents.

It was previously thought that SRB microorganisms could only survive in anoxic environments. However, studies over the past twenty years have revealed that some SRB are abundant in the surface layers of microbial mats that are oxygenated. This adaptation is likely achieved through the formation of close associations with other bacteria, such as sulfur-oxidizing bacteria. Sulfur-oxidizing bacteria can utilize the oxygen and produce the sulfide required for proliferation of SRB under seemingly unfavorable conditions [11,12,13,14]. This observation coupled with the wide metabolic diversity of SRB suggests their versatility in adapting to extreme pH and high temperature conditions, such as those present in seawater cooling tower systems and seawater injection pipelines used for oil recovery. SRBs are implicated as the main protagonists in the biocorrosion process, especially in the seawater environment where high concentration of sulfate allows growth of SRB within the biofilm structure [15].

Corrosion is usually a chemical process that involves the electrochemical oxidation of metal in the presence of oxygen and sulfur and can occur in both aerobic and anaerobic environments. For example, the chemical dissolution of iron results in the production of hydrogen (Equation (1)).

Fe + H^+ ^ = Fe^2+^ + H_2_ (1)

This phenomenon is often coupled with microbial induced corrosion (MIC). SRBs are considered to be the main cause of MIC. As SRB attach to the metal surface [16] (e.g., Fe) and reduce sulfate, oxygen is depleted quickly as a result of the heavily populated biofilm environment. This area of oxygen depletion becomes more anodic, and Fe^0^ acts as an electron donor to be oxidized to the soluble Fe^2+^ form, as shown in Equation (2):

4 Fe + SO_4_^2−^ + H_2_O = FeS + 3 Fe(OH)_2_ + 2 OH^−^ (2)

SRB consume H_2_ to reduce sulfate (Equation (3)) and influence the equilibrium of the chemical dissolution. In addition to this process, SRB are able to corrode iron surfaces through the formation of H_2_S, according to Equation (4) (chemical microbial induced corrosion, CMIC), and/or by directly using Fe as an electron donor (Fe^0^ = Fe^2+^ + 2e^−^). This process is called electrical microbial induced corrosion (EMIC) [17].

4 H_2_ + SO_4_^2−^ + H^+^ = HS^−^ + 4 H_2_O (3)

H_2_S + Fe^0^ = H_2_ + FeS (4)

A recent study has demonstrated that EMIC may play a more important role in biocorrosion by SRB when compared to CMIC. EMIC has thus far only been reported to be associated with the presence of *Desulfovibrionaceae* and *Desulfobulbaceae* families [6].

In addition to biocorrosion, SRB plays a role in the biofouling of the membranes used in seawater desalination treatment plants. The growth of sulfate reducers in the biofilms formed on RO membranes is enhanced by the high sulfate content of seawater and by the use of metabisulfite in the desalination process. Metabisulfite is commonly added to neutralize chlorine or other oxidizing biocides in desalination plants in order to protect the integrity of the membranes used. However, excess sulfate can select for SRB, which can produce corrosive hydrogen sulfide that damages membrane integrity [18].

### 2.2. Current Strategies to Tackle SRBs and Their Limitations

Biocides are commonly used to eliminate SRB by the following mechanisms: (i) disruption of membrane, envelope, capsid lipid, or protein constituents; (ii) blockage of receptor–ligand interactions; and (iii) inhibition of replication. Biocides can be either oxidizing or non-oxidizing agents. Oxidizing compounds (e.g., chlorine, bromine, ozone, and hydrogen peroxide) use radical substitution for the oxidation of organic compounds; electrophiles (e.g., formaldehyde, formaldehyde-releasing substances, isothiazolones) target cell walls and denature the amino groups of proteins; quaternary ammonium compounds (QUATS) dissolve and destabilize the cell membranes; and protonophores (e.g., parabens and weak acids like benzoic acid) acidify the cytoplasm of the cells to disrupt cellular metabolism [19]. Other common non-oxidizing biocides are methylene-bisthiocyanate (MTB) and tetrakis hydroxymethyl phosphonium sulfate (THPS) [1,20]. Different types of surfactants have also been used to tackle sulfate reducers in the oil and gas industry. Table 2 presents a summary of the biocides and surfactants commonly exploited to reduce unwanted microorganisms.

The use of biocides can have numerous problems. First, biocides like chlorine can result in the formation of carcinogenic and toxic disinfection byproducts, which impose a health, safety, and environment (HSE) concern. Second, it is known that SRB can produce extracellular polymeric substances (EPS), which, besides assisting in calcium precipitation and mineralization [31], also serve to embed this bacteria within the biofilm matrix. SRB has been reported to exist in biofilm matrices that are electroactive [32] or are determined to impart localized biocorrosion [2,33,34]. The biofilm matrix not only serves to adhere SRB onto surfaces but also hinders penetration of biocides. This, in turn, protects SRB from exposure to toxic biocides. Long-term exposure to sub-lethal concentrations of biocides has been demonstrated to select for resistant variants of other bacteria, and the possibility that this would occur for SRB cannot be excluded. The same concern is also applicable when alternative methods, otherwise termed as green approaches, are used. Although green approaches like the use of natural compounds including lemongrass essential oil, citrus, and cow urine (Table 2) would impose a lower HSE concern, most of these natural compounds demonstrate limited efficacies in field studies.

### 2.3. Biofilm Formation by SRB

Besides limiting the penetration and dissemination of biocides, biofilms are also intricately linked to biocorrosion. The presence of biofilm increases the rate of corrosion by up to 10,000 times, compared with the planktonic state [35]. This is likely due to poor oxygen diffusion throughout the biofilm that results in anoxic zones, in turn favoring SRB growth. The presence of SRB in the biofilm structure subsequently leads to localized biocorrosion events [36,37,38]. Furthermore, attachment on surfaces as facilitated by biofilm formation would allow direct contact between outer membrane proteins or electro-conductive nanowires and the metal surface [39,40].

To fully understand the contribution of SRB to biocorrosion and biofilm formation, a complete understanding of the biological mechanisms at the gene level is necessary. Recent genomic studies have revealed the nature of the genes that encode for the proteins required in sulfate reduction. A comprehensive review of the genes is out of the scope of this review, and interested readers should refer to several recent reviews related to this topic [41,42,43,44,45]. Instead, a brief summary of the genes related to biofilm formation is provided in this review.

Caffrey and co-workers utilized a transcriptomic approach to show that *Desulfovibrio vulgaris* biofilms upregulate many genes for flagellar components or proteins involved in flagellar biosynthesis to promote the switch from planktonic lifestyle to biofilm formation on an iron electrode [46]. Both flagella and pili are extracellular filaments attached to the cellular membrane. It is commonly thought that the main function of the flagella is to support rapid swimming motility, which, in the case of the study conducted by Caffrey et al., may imply the swimming motility towards an iron electrode and thereafter facilitating the attachment onto the electrode surface. Another study demonstrates that mutants of *Desulfovibrio alaskensis* lacking genes required for glycosytransferase, the pilus assembly protein, and the flagellar biosynthesis protein showed reduced biofilm formation and were unable to establish syntrophic cell-to-cell interactions with a partnering methanogen [47]. On the contrary, Zhang and colleagues found that, for the same bacterium, genes associated with flagellar motility were down-regulated among cells in the biofilm phase when compared with the planktonic state [48]. The discrepancy between these observations could be due to the differences in the level of biofilm maturation prior to sampling in both studies. In the latter study by Zhang et al., biofilm-associated cells were sampled at day 26 (i.e., early phase of biofilm formation) while a longer culturing time was taken by Krumholz et al., to establish the syntrophic interactions (i.e., late phase biofilm formation). Hence, the expression of flagella-associated genes in an established biofilm may be more distinct from that obtained during the early phase of biofilm formation.

Although no reports of nanowire or pili structures have been reported for SRB, it was observed that some SRB species possess organic filaments similar to nanowires [2], which may play a role in adhesion and biofilm formation. In addition, it was shown that the nanowire-like structures from SRB cells play an important role in direct interspecies electron transfer [49]. Likewise, extracellular electron transfer is known to occur in the presence of conductive nanowires or pili [33]. Conductive nanowires have been well studied in *Geobacter sulfurreducens.* It was previously observed that a pilus-deficient mutant was able to attach to an iron surface but could not reduce Fe(III) [50]. *Shewanella oneidensis* mutants deficient in *mtrC* and *omcA* genes (c-type cytochromes) and a functional Type II secretion system have pili, but exhibited poor conductivity [51]. These *Shewanella oneidensis* mutants were also unable to reduce hydrous ferric oxide. These combined observations exemplify the essential roles nanowires, pilis or flagellum-like appendages, c-type cytochromes, and type II secretion systems can play—not only in biofilm adherence and formation, but also in direct electron transfer and in metal reduction.

In addition, it was observed that biofilm-associated *D. vulgaris* cells, when compared to a planktonic batch culture, also demonstrate upregulation of echEF (subunits of Ech hydrogenase) and cytochrome c533 [52]. Ech catalyzes the reduction of ferredoxin with hydrogen. Ferredoxin proteins are, in turn, involved in the electrons transfer within the cell and are closely related with diverse protein complexes, such as the heterodisulfide reductase HdrABC [44].

The close interconnection between biofilm formation and extracellular electron transfer in SRBs would mean that biofilm formation by SRBs may play a crucial role in triggering biocorrosion events.

### 2.4. Biofilm Formation: the Role of QS and the Possible Link to Biocorrosion by SRB

Once a biofilm is established, the communication within the bacterial congregation allows them to synergistically perform certain phenotypic traits [53]. This communication is often termed as quorum sensing (QS), and is based on a set of small signal molecules (e.g., autoinducer-1, autoinducer-2, and peptides) produced and received by the microorganisms within the congregated mass. When the density of microbial cells increases, the density of different signal molecules correspondingly increases, and the different autoinducers bind to the receptors to activate or inactivate gene cascades [54]. Different bacteria use different signal molecules for QS, but the conventional notion is that gram-negative bacteria use autoinducer-1 (AI-1), otherwise referred to as acyl-homoserine lactone (acyl-HSL), while gram-positive bacteria use peptides as signaling molecules. In addition, autoinducer-2 (AI-2) or furanosyl borate diester [55] is commonly thought to be the universal signal molecule for interspecies communication. However, the role of AI-2 signaling and the presence of AI-2 related genes in QS have been called into question given that LuxS also plays a role in the activated methyl cycle [56,57]. The activated methyl cycle is an important metabolic pathway for the recycling of S-adenosylmethionine (SAM), the main methyl donor in methylation reactions required for cell growth, cell development, and chemotaxis. AI-2 is a product of SAM metabolism and may be indicative of the metabolic status of the cell and not present solely due to QS activity. Nevertheless, recent studies have shown that enzymes with AI-2 quenching capabilities have resulted in a significantly thinner biofilm that is more compact with a lower overall biovolume [58]. This finding suggests that, despite AI-2 having dual roles in both QS and metabolic pathways, quenching AI-2 may still be a good method to deter growth of unwanted bacterial populations.

Although the simplest QS system known is the *Vibrio fischieri* AHL-based LuxI/R system, the most well-studied model for QS is *Vibrio harveyi*. The system of this Gram-negative bacterium consists of three circuits based on three different signals [59]. In the first circuit, LuxM produces an AHL signal, termed as HAI-1, and binds to the sensor histidine kinase, LuxN. The second circuit is based on the AI-2 signal; this molecule is a furanosyl borate diester, produced by the synthase, LuxS. AI-2 is recognized by the LuxP protein in the periplasm. The LuxP/AI-2 complex interacts with another sensor histidine kinase, termed LuxQ. The third circuit involves a long-chain amino ketone, termed CAI-1, as the signal molecule. CqsA produces this signal molecule, which subsequently interacts with CqsS, a sensor histidine kinase. The expression of QS-related genes depends on the *luxR* gene. At low cell density, a phosphate group is transferred from the three autoinducers to the regulation protein, LuxO, within a cascade that also includes the protein, LuxU. In this state, LuxO is able to interfere with the *luxR* gene activity. In contrast, at high cell density, the signal receptors bind to the three different autoinducers, switching from kinase to phosphatase; the phosphate group is removed from LuxO. In this state, LuxO is not able to destabilize the *luxR* mRNA, hence resulting in a consequential expression of target genes.

Examples of target genes that can result in important phenotypic traits include those involved in biofilm formation. It was observed that biofilm formation in *Burkholderia cenocepacia* is regulated by the AtsR QS system [60], while in *Staphylococcus aureus* mutants of the Agr QS system produce more adhesins than the wild type due to the elimination of the Agr QS system that represses surface adhesins. Surface adhesins, in turn, mediate contact with surfaces [61,62,63]. In *Burkholderia glumae*, the TofR QS regulator is activated by the long chain C8-HSL autoinducer. It was demonstrated that a mutant deficient in *tofl*, and hence unable to receive QS signals, was unable to produce rhamnolipds and showed impaired swarming motility despite the presence of flagella [64]. Similarly, under phosphate-limited conditions, the Rhl QS system in *Pseudomonas aeruginosa* is upregulated to promote the hyperproduction of rhamnolipids so as to induce swarming motility [65].

Besides regulating production of rhamnolipids to enhance swarming motilities, QS influences the expression of genes related to flagella and pili synthesis, as demonstrated in *Sinorhizobium fredii* [66] and *Burkholderia glumae* [67]. There was also QS-dependent regulation of different types of secretion systems, such as the type VI secretion system in *Burkholderia glumae* [67], type III secretion systems in *Escherichia coli* [68] and *Aeromonas hydrophila* [69], and type II secretion proteins in *Pseudomonas aeruginosa* [70]. In these opportunistic pathogens, the secretion systems are most probably required for host invasion and toxin injection. However, as the earlier section has demonstrated links between flagella or type secretion systems with extracellular electron transfer, these reports infer a possible direct or indirect role QS may play in electron transfer. This is further supported by an observation in *P. aeruginosa,* in which spiking of C4 and C8-HSL showed an activation of numerous genes associated with c-type cytochromes. These proteins are important for the transfer of electrons and hence likely contribute to biocorrosion [70].

### 2.5. QS in SRBs: What is Known Thus Far?

Despite the tremendous wealth of knowledge related to QS in model bacterium species like *Vibrio harveyi* and *Vibrio fischeri*, this mechanism is still relatively unknown in SRBs although inferences on the presence of putative QS systems in SRBs can be made.

For example, a cell-free assay based on the expression of β-galactosidase was developed for rapid identification of QS activity in bacteria. The result demonstrates that several AHLs (C6 -AHL, *oxo*-C6 -AHL, C8-AHL, C10-AHL, and C12-AHL) were produced by *Desulfovibrio vulgaris* and other *Desulfovibrio* species [71,72]. Diverse AHLs were also detected from microbial mats containing a high abundance of SRBs [12]. Preliminary studies by Montgomery and coworkers suggest that AHLs with long chains of alkyl groups are more stable than short chain AHLs at elevated pH (>8.2), and that these long chain alkyl AHLs were produced by SRBs to stimulate sulfide oxidation by sulfide-oxidizing bacteria (SOB) [73]. It remains unknown if this cross-communication between SRB and SOB are a result of altruistic behavior from SRB to achieve co-existence of both microbial groups in the same microbial mat or because there is an underlying incentive for SRB to gain access to sulfate through SOBs.

In other studies, proteins homologous to LuxR and LuxS were found in *Desulfovibrio magneticus* [74], *D. desulfuricans* [75], and *D. vulgaris* [76]. A further mining of the SRB genomes available in the NCBI database also showed that proteins homologous to LuxS AI-2 synthase and the AI-2 receptors (i.e., LuxP and LuxQ) are present in many *Desulfovibrio* species. In addition, gene sequences homologous to that of LuxO, which is involved in the phosphorylation cascade to upregulate or repress quorum sensing associated genes, are also present in many *Desulfovibrio* species (Table 3). Based on these data mining results, as shown in Table 3, the presence of QS systems similar to that of *Vibrio harveyi* in several *Desulfovibrio* spp. can be inferred, specifically in *Desulfovibrio* *hydrothermalis* (with potential homologous proteins related to LuxS, LuxP, LuxQ, and LuxO) and *D. salexigens* (with potential homologous proteins related to LuxS, LuxQ, and LuxO).

Although gram-negative *Desulfovibrio* spp. is relatively better studied than other types of SRB, many SRBs that are present in seawater cooling tower systems or in the oil field injection systems are not *Desulfovibrio* spp., but instead belong to the thermophilic gram-positive *Desulfotomaculum* spp. [77] or other gram-negative bacteria like *Desulfonauticus* spp. [78]. Similar data mining in gram-positive *Desulfotomaculum* genomes showed that the majority of them (e.g., *Desulfotomaculum kuznetsovii*, *Desulfotomaculum ruminis*, *Desulfotomaculum reducens*, and *Desulfotomaculm acetoxidans*) have LuxR-type transcriptional regulator proteins. Given the lack of synthases discovered from genome-mining of SRBs, these LuxR-type proteins may simply be orphan receptors which have no signal to quench and/or to respond to and hence are not involved in QS. However, there also exists the possibility that these orphan receptors might allow the SRB to sense and respond to signals produced by other bacteria within a microbial community, or environmentally-modified autoinducers could bind to these orphan receptors so as to provide a broader environmental sensor [14]. Further studies are needed to determine what the downstream genes coordinated by the LuxR-type transcriptional regulators in SRB are, and whether there is a direct link between QS in SRB and sulfate-reduction and biocorrosion.

Similarly, non-*Desulfovibrio* gram-negative SRBs (e.g., *Desulfobacter postgatei*, *Desulfobacterium autotrophicum*, *Desulfobulbus propionicus*) are positive for AI-2 family transporters. However, all instances of genome mining showed no synthases or receptors homologous to the conventional ones found in *V. fischeri*. Nonetheless, it may be possible that synthase or receptor proteins different from those conventionally seen in *Vibrio* spp. may be present in these SRBs.

As mentioned in the earlier section, quorum-controlled genes have been identified in various pathogens, such as *Yersinia pestis* and *Pseudomonas aeruginosa* [65,79]. This has been done through the use of RNA-seq and genetic manipulations to derive mutant strains, and such approaches can be used on the SRBs to elucidate the quorum-controlled genes. Insights from the use of these approaches would serve to establish a relationship among QS, sulfate reduction, and biocorrosion. To elaborate, Schuster and coworkers utilized mutant strains of *Pseudomonas aeruginosa* PAO1 that are devoid of AHL receptors, comparing their gene expression profiles in the presence of HSL against those of the wild-type strain to determine the presence of quorum-activated genes. Similarly, *P. aeruginosa* PAO1 devoid of AHL synthases was determined for its quorum-activated genes when AHL was spiked during growth. Hence, using a similar approach and with *Desulfovibrio hydrothermalis* as a model bacterium, the identified LuxS, LuxP, and LuxQ in this SRB can be possibly inactivated or knocked out for comparison against the wild-type strain.

### 2.6. QQ as a Potential Green Biocidal Approach to Tackle QS

Given the importance of QS in various bacteria, including possibly SRBs, and that conventional biocides as highlighted in Table 2 can impose various detrimental effects, alternative green approaches should be explored. Recently a novel method, termed quorum quenching (QQ), has been demonstrated to reduce membrane biofouling and to repress the virulence factors of some pathogens by blocking the ability of communication in bacteria [80,81,82,83]. Likewise, the basis behind the QQ approach as a means to limit SRB is based on the assumption that interference of the communication channels would reduce biofilm formation and the associated metabolism of SRBs. Alternatively, QQ can also serve to deter the growth of other bacterial populations that may be supporting the proliferation of SRBs (e.g., aerobic microorganisms that help to establish an anoxic niche for SRB). However, it is important to note that a possible application of QQ to inhibit SRBs has not yet been exploited. This is likely due to the lack of understanding on whether QS is indeed present among SRBs.

Regardless, QQ can be achieved in three ways: (i) blocking synthesis of autoinducers; (ii) interfering with signal receptors; and (iii) degrading the autoinducers [84]. The first approach has been studied in a number of instances but not widely used [85,86,87], as blocking the synthesis of autoinducers in vivo within the cells is difficult to achieve. The majority of the QQ effort thus has been focused on either the second or third approach. For the second approach, compounds that have been recognized to interfere with signal receptors due to their structural similarity with the autoinducer have been identified. To illustrate, halogenated furanone secreted by *Delisea pulchra* is able to inhibit *Pseudomonas aeruginosa* QS by repressing the *las*B gene. The furanone compound acts as an analog to the cognate signal molecules and competitively binds onto receptors [88].

Alternatively, quorum-sensing antagonists of either natural or synthetic origin (e.g., vanillin [89], malic and lactic acids [90], or numerous biosurfactants [91]) are able to interfere with the receptor-binding process. Some of these compounds are produced by thermophiles and halophiles and hence make them suitable for application in seawater cooling tower systems and seawater injection systems. Both systems face unique and challenging environmental factors. These environmental factors include high salinity (of up to 58 g/L), high temperature (of up to 45 °C), and an alkaline pH of 7.2–8.0 [92], which serve to increase the technical complexity involved in developing QQ approaches to scavenge QS molecules.

Table 4 presents a non-exhaustive list of quorum sensing inhibitors with demonstrated quorum quenching effect in thermophilic or halophilic conditions. A more comprehensive list of small molecule compounds is also reviewed by Galloway and coworkers, although those compounds have not been tested for quenching effects in thermophilic or halophilic conditions [93].

Besides small molecules, quorum quenching can also be attained via enzymes. There are at least three known classes of enzymes for AHL degradation: (i) AHL-lactonase, (ii) AHL-acylase, and (iii) AHL oxidoreductases [114]. AHL-lactonase induces hydrolysis of the homoserine lactone ring while AHL-acylase hydrolyses the amide bond between the acyl side chain and the homoserine lactone in the AHL molecules. AHL-oxidoreductases reduce homoserine lactones, which then become unrecognizable by the receptors. AHL-lactonases and AHL-acylases originating from bacteria, and demonstrated to either exhibit activity or remain stable in high temperature or saline conditions are listed in Table 5.

As compared to that of AHL, enzyme-based QQ for AI-2 is not well studied. The only known enzyme to act on AI-2 is identified to be the AI-2 kinase (LsrK) from *E. coli.* This enzyme degrades AI-2 and has been demonstrated to reduce the QS response of *Salmonella* Typhimurium and *Vibrio harveyi* [115]. AI-2 kinase, however, requires the presence of ATP, which decomposes at acidic pH and/or at elevated temperature [114], thus limiting the applicability of this enzyme in seawater cooling tower and injection systems.

Recently, by constructing large-insert cosmid libraries from metagenomes derived from various saline samples, enzymes displaying homologies to oxidoreductases, proteases, amidases, and aminotransferases were identified to exhibit a QQ effect against both AHL and AI-2 [58]. To exemplify, enzymes ranging from 177–478 aa that share close homology (42%–100% aa identity) with either aminotransferase, 3-hydroxy-2-methylbutyry-CoA dehydrogenase, ferredoxin reductase, 4-hydroxy-3-methybut-2-en-1-yl diphosphate synthase, 3-beta hydroxysteroid dehydrogenase, or N-acetylmuramoyl-L-alanine amidase were recovered from the Black Sea and salt marsh. This finding suggests that the types of enzymes that can quench AI-2 extend beyond the AI-2 kinase.

### 2.7. Potential Strategies for QQ Application to Tackle SRB

Despite the advantages associated with enzyme-based QQ approaches, the application of QQ can be technically challenging. This is particularly the case in harsh environmental conditions representative of seawater cooling towers or injection systems. As mentioned earlier, both systems face challenging environmental factors, including high salinity of up to 58 g/L, high temperature of up to 45 °C, and an alkaline pH of 7.2 to 8.0 [92]. Therefore, although a considerable number of QQ enzymes and QSIs have been screened and characterized, as detailed in Table 4 and Table 5, it is feasibly challenging to adapt them for industrial applications.

Several strategies can be applied to overcome these technical challenges. The first step would involve screening for the novel genes responsible for the thermostable QQ enzymes. The sources of such genes potentially include extremophiles, as detailed in Table 5. Bacterial candidates include *Geobacillus caldosilitycus* YS-8, which is able to degrade N-acylhomoserine lactone at temperatures from 30 to 70 °C, with a maximum activity at 50 °C and pH 7.5 [102]. It has been reported that the gene, *aii*A, from *Bacillus* sp. strain AI96, encodes for a thermostable AHL lactonase [106]. This enzyme is stable at 80 °C and shows full activity from 10 to 40 °C at pH 8.0. Thermostable AHL lactonase enzymes with maximum activity at high temperature have also been isolated from *Tenacibaculum* sp. 20J [108] and *Thermaerobacter marianensis* [109], both of which are marine bacteria. These can be potential candidates for the isolation of related genes that express useful thermostable QQ enzymes. Protein engineering methods such as site mutagenesis or directed evolution can also be applied to modify the properties of QQ enzymes to tailor for their use under specific conditions.

Once the appropriate gene has been identified, the gene can be manipulated into a genetic system to overexpress the associated QQ enzymes. For instance, *Bacillus megaterium* can be selected as a potential genetic system for this experiment because it is resistant at the temperature, salinity, and pH conditions mentioned above. Furthermore, expression vectors are already developed for and stable in this bacterium. At the same time, the bacterial host should also be chosen based on its ability to express extracellular QQ enzymes in larger quantities compared to the wild-type strains. This would enable the direct application of the bacterium in the system, circumventing the need to operate separate bioreactors for production and purification of the QQ enzymes.

A genetic system capable of overexpressing the QQ enzymes can be directly applied to a relevant system via different strategies. Immobilization or encapsulation of bacterial hosts secreting QQ enzymes in magnetic carriers, beads, or hollow-fiber vessels has been previously applied to achieve lower membrane fouling in wastewater treatment systems [84,116,117]. It is proposed that such a strategy would allow continuous growth and self-replenishment of the bacterial source and enzymes within the system. Furthermore, proper selection of the encapsulation material would also allow for the minimization of any potential toxic shock loading that may render the bacteria and QQ enzymes inactive.

The advantage of the quorum quenching approach is based on to the limited inclination of the target bacteria to develop resistant traits to its effects. In fact, quorum quenching agents do not affect bacterial growth, but only their ability to switch to a collective behavior. Despite having its advantages, Kalia et al., pointed out the possibility that bacteria can block the QS system at high concentrations of inhibitors and return to their natural condition when these concentrations decrease [118]. For this reason, it will be important to investigate bacterial behavior in the presence of a QS inhibitor to avoid the evolution of SRB resistant to QQ approaches. Furthermore, the QQ approach is not likely to be effective when directly applied to an open system. Diffusion of QQ enzymes or QS inhibitors would render a loss in the localized blocking of QS communications, and could limit their intended effect.

Instead, the QQ approach may be more feasible when applied to closed systems. To illustrate, it has been demonstrated in various lab-scale reactors that membrane biofouling can be controlled through the addition of quorum quenching enzymes or QS inhibitors. A corresponding improvement in the water flux and decrease in biofilm thickness were observed. A recent study involving pilot-scale MBRs (i.e., closed reactor systems) that were operated for at least four months to treat real-strength municipal wastewater [119,120] also showed that QQ was able to delay transmembrane pressure build-up and reduce EPS concentrations in membrane biofilms [83]. Although similar demonstrations of QQ to reduce SRBs are not available, these findings collectively show the potential of a QQ approach to delay the biofilm formation process and hence avoid the consequential effect of biocorrosion in a contained system (e.g. seawater injection pipeline or the seawater cooling tower).

## 3. Conclusions

This review addresses the need and basis for developing new and green approaches to reducing SRB in engineered industrial systems, primarily due to their associated roles in biofilm formation and subsequent effect on biocorrosion. Rampant use of the conventionally deployed antibiotics and biocides are ineffective long-term solutions to SRB, as evidenced by the emergence of antibiotic-resistant bacteria and antibiotic-resistance genes in many other types of bacteria. The continued application of biocides against microorganisms involved in the biocorrosion process also places an unsustainable pressure on the environment when these compounds are unintentionally disseminated into surrounding ecosystems. The quorum quenching approach, as discussed in this review, would serve as an environmentally friendly alternative. However, prior to exploiting the quorum quenching approaches outlined in this study to tackle the biocorrosion process, future studies should focus on understanding the quorum sensing mechanisms in SRB. Despite the evidence of a putative quorum sensing system in many SRB, a more complete picture of the genes controlled by QS and the genes machinery involved is necessary to develop a successful QQ approach.

## Figures and Tables

**Table 1:** Redox reactions for the formation of hydrogen sulfide by sulfate-reducing bacteria in the presence of exemplary electron donors and electron acceptors.

4 H_2_ + SO_4_^2−^ + H^+^ = HS^−^ + 4 H_2_O	ΔG^0′^ (KJ/rx) = −151.9
CH_3_COO^−^ + SO_4_^2−^ = 2 HCO_3_^−^ + HS^−^	ΔG^0′^ (KJ/rx) = −47.6
CH_3_CH_2_COO^−^ +0.75 SO_4_^2−^ = CH_3_COO^−^ + HCO_3_^−^ 0.75 HS^−^ + 0.25 H^+^	ΔG^0′^ (KJ/rx) = −37.7
CH₃CH₂CH₂COO^−^ + 0.5 SO_4_^2−^ = 2 CH_3_COO^−^ + 0.5 HS^−^ + 0.5 H^+^	ΔG^0′^ (KJ/rx) = −27.8
CH_3_CHOHCOO^−^ + 0.5 SO_4_^2−^ = CH_3_COO^−^ + HCO_3_^−^ + 0.5 HS^−^	ΔG^0′^ (KJ/rx) = −80.2

**Table 2:** Commonly used reagents and their mode of action, required dosage and associated disadvantages.

Class	Biocide	Action	Dosage	Other Information	Ref.
Oxidizing biocides	Chlorine	Direct oxidation, destruction of the cell walls through modification of membrane permeability, leakage of cellular constituents, protein inactivation, damage of nucleic acid.	0.5 ppm	They have numerous disadvantages: (i) interaction with other chemicals to result in toxic disinfectant byproducts (ii) contribute to corrosion of structural metals (iii) weaken the integrity of non-metallic components (iv) ineffective against bacteria embedded within biofilm matrix.	[1,20]
	Bromine		0.05–0.1 ppm		
	Ozone		0.2–0.5 ppm		
	Hydrogen peroxide		50–100 ppm		
	Magnesium peroxide/ORC™		1%–2% MgO_2_: 1% MgO_2_ + 1% ORC		[21]
Non-oxidizing biocides	Glutaraldehyde	Reacts with proteins on the cell membrane and cytoplasm.	10–70 ppm	Generally toxic and persistent in the environment into which they are being discarded. To reduce the dosage, in the recent past they have been tested in a cocktail with 1000-2000 ppm Ethylenediaminedisuccinate (EDDS), a chelator that increases the permeability of membranes by chelating with Mg^2+^ and Ca^2+^, and methanol or ethanol that denature the proteins of the outer membrane [22,23]. The use of ultrasound was also able to increase their efficacy [24].	[1,20]
	QUATS (Quaternary ammonium compounds)	Impose detergent effect on cell, dissolute lipids and thus cause loss of cellular content.	8–35 ppm		
	Isothiazolones	Exhibit cytotoxicity on different types of cells.	0.9–10 ppm		
	MTB (Methylene-bisthiocyanate)	Prevents cell growth by blocking essential chemical reactions that occur within the cell.	1.5–8 ppm		
	THPS (tetrakishydroxymethyl phosphonium sulfate)	Cytotoxic effect, with loss of membrane integrity. Mainly used in water treatment systems and oil field operations. Low environmental toxicity.	10–90 ppm		
Natural biocides	Lemongrass essential oil and citrus	Antimicrobial effect due to membrane alteration and formation of electron-dense inclusions. Loss of ions and reduction of membrane potential will occur.	0.17–0.84 ppm	Showed limited efficacies in large-scale operations.	[25]
	Cow Urine	Reduces the planktonic and biofilm population in the same way. A reduction of sulfide, Fe(III), and EPS production was observed.	25 ppm		[26]
Surfactants	Imidazolium-based Gemini Surfactants	Amphiphilic molecules create a biomolecular layer on the metal surface. Also, hydrophobic chains of surfactants can penetrate through bacterial cell membranes, leading to strong bacterial damage. Shchiff bases are usually used to synthesize other antibacterial compounds.	5000 ppm	Applied in the oil and gas industry to reduce the action of SRB to delay the biocorrosion process.	[27]
	Phosphonium Surfactant compounds		50–400 ppm for 3 h		[28]
	Cationic surfactants based on Schiff bases		20–400 ppm on cultured media		[29]
	Gemini Surfactant	Forms a protective film on the surface. Electrostatic interaction between the negatively charged cell membrane (lipoprotein) and the positively charged ammonium groups of the synthesized gemini surfactant. Moreover, physical disruption of the bacterial cell membrane takes place when the surfactant’s alkyl hydrophobic chain penetrates into the bacterial cell membrane.	1 mM		[30]

**Table 3:** QS (quorum sensing) protein homologs in SRB (sulfate-reducing bacteria). Each protein was compared with the homologue in *Vibrio harveyi.*

Protein	SRB	Best Matched Protein Name in Database that was Homologous to the Listed QS Protein	Amino Acids Identity %	Query Cover %	E Value
LuxS	*Desulfovibrio hydrothermalis*	S-ribosylhomocysteine lyase	33	100	3E-19
	*Desulfovibrio salexigens*	Quorum sensing AI-2, LuxS	34	100	2E-18
	*Desulfotalea psychrophila*	Probable q. s. AI-2 production protein, LuxS	34	85	4E-16
LuxR	*Desulfovibrio desulfuricans*	2 components transcriptional regulator	38	99	4E-47
	*Desulfovibrio africanus*	Transcriptional regulator, LuxR family	31	98	7E-21
	*Desulfovibrio africanus*	2 components transcriptional regulator	32	97	2E-34
	*Desulfovibrio magneticus RS-1*	LuxR family transcriptional regulator	48	34	1E-17
	*Desulfotomaculum nigrificans*	2 components transcriptional regulator, LuxR family	35	99	5E-42
	*Desulfotomaculum acetoxidans*	2 components transcriptional regulator, LuxR family	24	94	3E-08
	*Desulfotomaculum acetoxidans*	Transcriptional regulator, LuxR family	27	94	1E-15
	*Desulfotomaculum kuznetsovii*	2 components transcriptional regulator, LuxR family	38	99	3E-49
	*Desulfotomaculum reducens*	2 components transcriptional regulator, LuxR family	38	99	3E-41
	*Desulfotomaculum ruminis*	Regulatory protein, LuxR	52	16	2E-11
	*Desulfosarcina cetonica*	LuxR family transcriptional regulator	26	90	6E-16
	*Desulfobacterium autotrophicum*	2 components transcriptional regulator, LuxR family	39	98	2E-43
	*Desulfobacterium autotrophicum*	LuxR family transcriptional regulator	47	17	2E-05
	*Desulfobulbus propionicus DSM 2032*	2 components transcriptional regulator, LuxR family	34	98	5E-41
	*Desulfovibrio vulgaris (str. Hildenborough)*	LuxR family transcriptional regulator	32	93	2E-29
	*Syntrophobacter fumaroxidans*	2 components transcriptional regulator, LuxR family	40	96	2e-51
	*Thermodesulfobium narugense*	2 components transcriptional regulator, LuxR family	33	90	4e-32
	*Thermodesulfovibrio aggregans*	LuxR family transcriptional regulator	35	99	3e-36
LuxP	*Desulfovibrio piezophilus*	AI-2 binding perisplatic protein, LuxP	42	95	7E-95
	*Desulfovibrio hydrothermalis*	AI-2 binding perisplatic protein, LuxP	43	94	6E-103
	*Desulfovibrio alaskensis*	AI-2 binding perisplatic protein, LuxP precursor	44	89	2E-105
LuxQ	*Desulfovibrio salexigens*	PAS/PAC sensor signal transduction histidine kinase	29	31	2E-22
	*Desulfovibrio hydrothermalis*	Signal transduction histidine kinase	31	31	5E-23
LuxO	*Desulfotignum phosphitoxidans*	Luminescence regulatory protein, LuxO	45	67	7E-85
	*Desulfovibrio salexigens*	PAS modulated sigma54 specific transcriptional	53	55	2E-83
	*Desulfovibrio magneticus RS-1*	Fis family transcriptional regulator	43	68	5E-75
	*Desulfovibrio vulgaris*	Sigma54 specific transcriptional regulator	46	53	4E-82
	*Desulfovibrio hydrothermalis*	PAS modulated sigma54 specific transcriptional	54	55	1E-84
	*Desulfovibrio africanus*	PAS modulated sigma54 specific transcriptional	39	69	4E-81
CqsS	*Desulfovibrio salexigens*	PAS/ signal transduction histidine kinase	34	52	1E-83
	*Desulfovibrio magneticus*	Multi-sensor hybrid histidine kinase	39	28	8E-77

**Table 4:** Natural or synthetic compounds with demonstrated quorum quenching effects in thermophilic or halophilic conditions. QSI denotes quorum sensing inhibitor.

Name	Origin	Structure	Action Mechanism	Treatment Condition	QSI Effect	Ref.
N (2′-phenylethyl)-Isobutyramide 3-methyl-N (2′-phenylethyl)-butyramide	*Halobacillus salinus* C42 (sea grass)	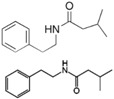	Analog: Competes with N-acyl homoserine lactones for receptor binding	30 °C in marine broth	QS system inhibition of AHL in biosensor *Chromobacterium violaceum*	[94]
Unknown compound; AHL QSI	*Favia sp. coral isolate Fav 2-50-7* (>98% similarity to *Vibrio harveyi*)		Possible AHL analog	Maintains anti-QS activity at high temperature (from 26 °C to 95 °C)	Antibiofilm of *P. aeruginosa* by QS inhibition	[95]
Penicillic acid	commercial	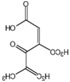	Autoinducer antagonist that may interfere with the stability and function of the autoinducer synthase or QS regulator protein	artificial seawater 30 °C	Inhibit AI-2 activity and biofilm formation of marine strain *Halomonas pacific*, QS inhibition dose is 25 µM concentration	[96]
Patulin	commercial	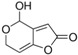				
Vanillin	commercial	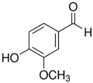	Interfere or modify the structure of AHL to hinder the binding of AHL to receptor protein	Seawater environment	Reduce seawater desalination RO membrane biofouling. Vanillin suppresses EPS production for various marine bacterial communities on the RO membrane surface, QS inhibition dose is 1200 mg/L	[89]
Cinnamaldehyde		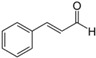	Reduce the DNA-binding ability of LuxR			
4-nitropyridine-N-oxide	Synthetic Compound		QSI analogue	Seawater environment	Inhibits the formation of diatom-biofilm caused by two marine diatoms *Cylinthrotheca sp*. and *Nitzschia closterium.* QS inhibition dose is 10 mg/L	[97]
Hexadecanoic acid	Marine cyanobac terium *Synechococcus* sp. Q-25		antagonistic binding to the AHL receptor protein	Marine LB broth (pH 7.5 ± 0.2) at 30 °C	Reduces the biofilm and EPS formation of marine infectious pathogens *Vibrio harveyi* and *Vibrio vulnificus*	[98]
Isonaamidine A	Marine sponge *Leucetta chagosensis*		AI-2 inhibitor	Artificial seawater	Inhibits strongly the AI-2 channel of *Vibrio harveyi*	[99]

**Table 5:** List of AHL-lactonases and AHL-acylases that exhibit activity under thermophilic or saline conditions.

Name	Origin	Property	Quenching Effect/Target	Ref.
AHL acylase	*Bacillus pumilus* S8-07 (Palk Bay)	Retains activity after incubation at 70 °C for 10 min.	Causes reduction of virulence factors and biofilm in *Pseudomonas aeruginosa* PAO1 and *Serratia marcescens*	[100]
AHL lactonase (AiiAB546)	*Bacillus* sp. B546 (mud of a fish pond)	Shows optimal activity at pH 8.0, 20 °C, stable at pH 8.0–12.0, however also remains thermostable at 70 °C and is highly resistant to proteases.	C10-HSL, C12-HSL, C6-HSL, 3-oxo-C6-HSL, 3-oxo-C8-HSL, C8-HSL Attenuates *Aeromonas hydrophila* infection in carp	[101]
AHL lactonase	*Geobacillus caldoxylosilyticus* YS-8, (volcano soil)	Exhibits activity over a wide temperature range of 30–70 °C, optimal temperature and pH: 50 °C and pH 7.5.	C6-HSL, 3-oxo-C12-HSL, 3-oxo-C6-AHL, C8-HSL	[102]
AHL lactonase (AiiA TSAWB)	*Bacillus* sp. TSAWB (salty soil)	Shows hydrolysis activity in presence of 0%–5% salinity.	C10-HSL	[103]
AHL lactonase (SisLac)	*Bacillus* sp. TSAWB (salty soil)	Optimal activity at pH 9.0, enzymatic half-life of 84 min at 85 °C.	C8-HSL, and C10-HSL	[104]
Phosphotriesterase-like lactonases (SsoPox)	Hyperthermophilic archaeon *Sulfolobus solfataricus* MT4	Exhibits activity over a broad pH range of 5.0–9.5, thermostable at 70 °C to 85 °C.	3-oxo-C8-HSL, 3-O-C6-HSL, C4-HSL	[105]
AHL lactonase (AiiAAI96)	*Bacillus* sp. AI96 (pond sediment)	Possesses high activity under broad conditions: ranging from pH 6.0 to 8.5 and 10 °C to 40 °C. Also stable at 70°C, pH 8.0 for at least 1 h.	C4-HSL, C6-HSL, C7-HSL, C8-HSL, C10-HSL, C12-HSL, C14-HSL, 3-oxo-C8-HSL, 3-oxo-C6-HSL, 3-oxo-C10-HSL, 3-oxo-C12-HSL, 3-oxo-C14-HSL, 3-hydroxy-C8-HSL, 3-hydroxy-C14-HSL. Attenuates *Aeromonas hydrophila* infection in zebrafish by oral feeding.	[106]
AHL lactonase (AiiA)	*Bacillus licheniformis* DAHB1	Optimal activity at pH: 7.0–8.0 and temperature range: 30–50 °C. Maintains 90% activity after incubation at 60 °C–80 °C for 1 h. Resistant to acidic environment and proteases.	C4-HSL, C6-HSL, 3-oxo-C6-HSL, C8-HSL, 3-oxo-C8-HSL, C10-HSL, C12-HSL, C14-HSL, Inhibits biofilm formation and viable counts of *Vibrio parahaemolyticus* and attenuates infection and mortality of shrimps in aquaculture	[107]
AHL lactonase (Aii20J)	Marine bacteria *Tenacibaculum* sp. strain 20 J	Crude enzyme stays active under 100 °C for 10 min, resistant to proteinase K and α-chymotrypsin, unaffected by wide ranges of pH.	C4-HSL, C6-HSL, C8-HSL, C10-HSL, C12-HSL, C14-HSL, 3-oxo-C6-HSL, 3-oxo-C12-HSL, 3-oxo-C10-HSL, 3-OH-C10-HSL, 3-oxo-C12-HSL, 3-OH-C12-HSL, 3-oxo-C13-HSL, 3-oxo-C14-HSL, Quenches AHL-mediated acid resistance in *Escherichia coli*	[108]
AHL lactonase (AiiT)	Marine bacteria *Thermaerobacter marianensis* JCM 10246	Shows AHL degradation activity at temperature ranging from 40 to 80°C. Maintains 80% of enzyme activity after incubation at 40, 60 and 70 °C for 10 min.	C6-HSL, C8-HSL, C10-HSL	[109]
AHL lactonase (QsdH)	*Pseudoalteromonas byunsanensis* strain 1A01261	Exhibits activity over a temperature range of 20–60 °C. Stays active after 60 °C for 30 min.	3-oxo-C8-HSL, 3-oxo-C6-HSL, C4-HSL, C6-HSL, C8-HSL, C10-HSL, C12-HSL, C14-HSL, Attenuates pathogenicity of plant pathogen *Erwinia carotovora* under 0.15 M NaCl	[110]
AHL lactonase (MomL)	*Muricauda olearia* Th120	Exhibits high activity range from 20–50 °C. Retains 30% activity after incubation at 60 °C for 30 min.	C6-HSL, C12-HSL, 3-oxo-C6HSL, C8-HSL, 3-oxo-C8-HSL, C4-HSL, 3-oxo-C10-HSL, C14-HSL, 3-oxo-C14-HSL, C10-HSL. Attenuates virulence of *Pseudomonas aeruginosa* and *Caenorhabditis elegans*	[111]
AHL lactonase	*Tenacibaculum soleae* T173	Maintains C6-HSL degrading activity after boiled for 30 min.	C6-HSL, 3-oxo-C6-HSL, C8-HSL,3-oxo-C8-HSL, C10-HSL, 3-oxo-C10-HSL, C12-HSL, 3-oxo-C12-HSL, C14-HSL and 3-oxo-C14-HSL	[112]
Phospshotriesterase-like Lactonase	*Geobacillus kaustophilus* HTA426	Retains its catalytic activity at 60 °C for up to 72 h.	C4-HSL, C6-HSL, 3-oxo-C6-HSL, C8-HSL, 3-oxo- C8-HSL, C10-HSL	[113]

## References

[1] Lin J., Ballim R. (2012). Biocorrosion control: Current strategies and promising alternatives. Afr. J. Biotechnol..

[2] AlAbbas F.M., Williamson C., Bhola S.M., Spear J.R., Olson D.L., Mishra B., Kakpovbia A.E. (2013). Influence of sulfate reducing bacterial biofilm on corrosion behavior of low-alloy, high-strength steel (api-5l x80). Int. Biodeter. Biodegr..

[3] Williamson R., Javaherdashti R., Tan H., Javaherdashti R., Nwaoha C., Tan H. (2013). Corrosion and Materials in the Oil and Gas Industries.

[4] NACE Corrosion costs and preventive strategies in the united states. https://www.nace.org/uploadedFiles/Publications/ccsupp.pdf.

[5] Heitz E., Flemming H.C., Sand W. (2012). Microbially Influenced Corrosion of Materials: Scientific and Engineering Aspects.

[6] Enning D., Garrelfs J. (2014). Corrosion of iron by sulfate-reducing bacteria: New views of an old problem. Appl. Environ. Microbiol..

[7] Sawford M.K., Ateya B.G., Abdullah A.M., Pickering H.W. (2002). The role of oxygen on the stability of crevice corrosion. J. Electrochem. Soc..

[8] Kahrilas G.A., Blotevogel J., Stewart P.S., Borch T. (2015). Biocides in hydraulic fracturing fluids: A critical review of their usage, mobility, degradation, and toxicity. Environ. Sci. Technol..

[9] OSHA Osha fact sheet formaldehyde. https://www.osha.gov/OshDoc/data_General_Facts/formaldehyde-factsheet.pdf.

[10] Muyzer G., Stams A.J.M. (2008). The ecology and biotechnology of sulphate-reducing bacteria. Nat. Rev. Microbiol..

[11] Baumgartner L.K., Reid R.P., Dupraz C., Decho A.W., Buckley D.H., Spear J.R., Przekop K.M., Visscher P.T. (2006). Sulfate reducing bacteria in microbial mats: Changing paradigms, new discoveries. Sediment. Geol..

[12] Decho A.W., Visscher P.T., Ferry J., Kawaguchi T., He L.J., Przekop K.M., Norman R.S., Reid R.P. (2009). Autoinducers extracted from microbial mats reveal a surprising diversity of N-acylhomoserine lactones (ahls) and abundance changes that may relate to diel pH. Environ. Microbiol..

[13] Jonkers H., Koh I.-O., Behrend P., Muyzer G., de Beer D. (2005). Aerobic organic carbon mineralization by sulfate-reducing bacteria in the oxygen-saturated photic zone of a hypersaline microbial mat. Microbiol. Ecol..

[14] Decho A.W., Norman R.S., Visscher P.T. (2010). Quorum sensing in natural environments: Emerging views from microbial mats. Trends Microbiol..

[15] Zhang C., Wen F., Cao Y. (2011). Progress in research of corrosion and protection by sulfate-reducing bacteria. Procedia Environ. Sci..

[16] Kuang F., Wang J., Yan L., Zhang D. (2007). Effects of sulfate-reducing bacteria on the corrosion behavior of carbon steel. Electrochim. Acta.

[17] Kato S. (2016). Microbial extracellular electron transfer and its relevance to iron corrosion. Microbial. Biotechnol..

[18] Hong P.-Y., Moosa N., Mink J. (2016). Dynamics of microbial communities in an integrated ultrafiltration–Reverse osmosis desalination pilot plant located at the arabian gulf. Desalin. Water Treat..

[19] Turkiewicz A., Brzeszcz J., Kapusta P. (2013). The application of biocides in the oil and gas industry. Nafta-Gaz.

[20] Videla H.A. (2002). Prevention and control of biocorrosion. Int. Biodeter. Biodegr..

[21] Chang Y.J., Chang Y.T., Hung C.H. (2008). The use of magnesium peroxide for the inhibition of sulfate-reducing bacteria under anoxic conditions. J. Ind. Microbiol. Biot..

[22] Wen J., Xu D., Gu T., Raad I. (2012). A green triple biocide cocktail consisting of a biocide, edds and methanol for the mitigation of planktonic and sessile sulfate-reducing bacteria. World J. Microbiol. Biot..

[23] Wen J., Zhao K.L., Gu T.Y., Raad I.I. (2009). A green biocide enhancer for the treatment of sulfate-reducing bacteria (SRB) biofilms on carbon steel surfaces using glutaraldehyde. Int. Biodeter. Biodegr..

[24] Rasol R.M., Noor N.M., Yahaya N., Abdullah A., Abu Bakar A., Rashid A.S.A. (2014). Combination effects of ultrasound wave and biocide treatment on the growth of sulfate reducing bacteria (SRB). Desalin. Water Treat..

[25] Korenblum E., Goulart F.R.D., Rodrigues I.D., Abreu F., Lins U., Alves P.B., Blank A.F., Valoni E., Sebastian G.V., Alviano D.S. (2013). Antimicrobial action and anti-corrosion effect against sulfate reducing bacteria by lemongrass (*Cymbopogon citratus*) essential oil and its major component, the citral. AMB Express.

[26] Lavania M., Sarma P.M., Mandal A.K., Cheema S., Lal B. (2011). Efficacy of natural biocide on control of microbial induced corrosion in oil pipelines mediated by *Desulfovibrio vulgaris* and *Desulfovibrio gigas*. J. Environ. Sci..

[27] Aiad I., Emam D., El-Deeb A., Abd-Alrahman E. (2013). Novel imidazolium-based gemini surfactants: Synthesis, surface properties, corrosion inhibition and biocidal activity against sulfate-reducing bacteria. J. Surfactants Deterg..

[28] Aiad I.A., Tawfik S.M., Shaban S.M., Abd-Elaal A.A., El-Shafie M. (2014). Enhancing of corrosion inhibition and the biocidal effect of phosphonium surfactant compounds for oil field equipment. J. Surfactants Deterg..

[29] Shaban S.M., Saied A., Tawfik S.M., Abd-Elaal A., Aiad I. (2013). Corrosion inhibition and biocidal effect of some cationic surfactants based on schiff base. J. Ind. Eng. Chem..

[30] Labena A., Hegazy M.A., Horn H., Muller E. (2015). The biocidal effect of a novel synthesized gemini surfactant on environmental sulfidogenic bacteria: Planktonic cells and biofilms. Mater. Sci. Eng..

[31] Braissant O., Decho A.W., Dupraz C., Glunk C., Przekop K.M., Visscher P.T. (2007). Exopolymeric substances of sulfate-reducing bacteria: Interactions with calcium at alkaline pH and implication for formation of carbonate minerals. Geobiology.

[32] Cordas C.M., Guerra L.T., Xavier C., Moura J.J. (2008). Electroactive biofilms of sulphate reducing bacteria. Electrochim. Acta.

[33] Li H.B., Xu D.K., Li Y.C., Feng H., Liu Z.Y., Li X.G., Gu T.Y., Yang K. (2015). Extracellular electron transfer is a bottleneck in the microbiologically influenced corrosion of C1018 carbon steel by the biofilm of sulfate-reducing bacterium *Desulfovibrio vulgaris*. PLoS ONE.

[34] Wikieł A.J., Datsenko I., Vera M., Sand W. (2014). Impact of *Desulfovibrio alaskensis* biofilms on corrosion behaviour of carbon steel in marine environment. Bioelectrochemistry.

[35] Videla H.C.A. (1996). Manual of Biocorrosion.

[36] Duan D.X., Lin C.G. (2011). Effect of surface free energy and electrochemical polarization on attachment of sulfate reducing bacteria. Adv. Mater. Res..

[37] Elmouaden K., Jodeh S., Chaouay A., Oukhrib R., Salghi R., Bazzi L., Hilali M. (2016). Sulfate-reducing bacteria impact on copper corrosion behavior in natural seawater environment. J. Surf. Eng. Mater. Adv. Technol..

[38] Beech I.B., Sunner J.A., Hiraoka K. (2005). Microbe-surface interactions in biofouling and biocorrosion processes. Int. Microbiol..

[39] Lovley D.R. (2011). Live wires: Direct extracellular electron exchange for bioenergy and the bioremediation of energy-related contamination. Energ. Environ. Sci..

[40] Xu D., Li Y., Gu T. (2016). Mechanistic modeling of biocorrosion caused by biofilms of sulfate reducing bacteria and acid producing bacteria. Bioelectrochemistry.

[41] Zhou J., He Q., Hemme C.L., Mukhopadhyay A., Hillesland K., Zhou A., He Z., van Nostrand J.D., Hazen T.C., Stahl D.A. (2011). How sulphate-reducing microorganisms cope with stress: Lessons from systems biology. Nat. Rev. Microbiol..

[42] Wall J., Bill Yen H., Drury E., Barton L., Hamilton W. (2007). Evaluation of Stress Response in Sulphate-Reducing Bacteria through Genome Analysis.

[43] Keller K.L., Wall J.D. (2011). Genetics and molecular biology of the electron flow for sulfate respiration in *Desulfovibrio*. Front. Microbiol..

[44] Pereira I.A.C., Ramos A.R., Grein F., Marques M.C., da Silva S.M., Venceslau S.S. (2011). A comparative genomic analysis of energy metabolism in sulfate reducing bacteria and archaea. Front. Microbiol..

[45] Price M.N., Ray J., Wetmore K.M., Kuehl J.V., Bauer S., Deutschbauer A.M., Arkin A.P. (2014). The genetic basis of energy conservation in the sulfate-reducing bacterium *Desulfovibrio alaskensis* g20. Front. Microbiol.

[46] Caffrey S.M., Park H.S., Been J., Gordon P., Sensen C.W., Voordouw G. (2008). Gene expression by the sulfate-reducing bacterium *Desulfovibrio vulgaris* hildenborough grown on an iron electrode under cathodic protection conditions. Appl. Environ. Microbiol..

[47] Krumholz L.R., Bradstock P., Sheik C.S., Diao Y., Gazioglu O., Gorby Y., McInerney M.J. (2015). Syntrophic growth of *Desulfovibrio alaskensis* requires genes for H2 and formate metabolism as well as those for flagellum and biofilm formation. Appl. Environ. Microbiol..

[48] Zhang W., Culley D.E., Nie L., Scholten J.C. (2007). Comparative transcriptome analysis of *Desulfovibrio vulgaris* grown in planktonic culture and mature biofilm on a steel surface. Appl. Microbiol. Biotechnol..

[49] Wegener G., Krukenberg V., Riedel D., Tegetmeyer H.E., Boetius A. (2015). Intercellular wiring enables electron transfer between methanotrophic archaea and bacteria. Nature.

[50] Reguera G., McCarthy K.D., Mehta T., Nicoll J.S., Tuominen M.T., Lovley D.R. (2005). Extracellular electron transfer via microbial nanowires. Nature.

[51] Gorby Y.A., Yanina S., McLean J.S., Rosso K.M., Moyles D., Dohnalkova A., Beveridge T.J., Chang I.S., Kim B.H., Kim K.S. (2006). Electrically conductive bacterial nanowires produced by *Shewanella oneidensis* strain MR-1 and other microorganisms. Proc. Natl. Acad. Sci. USA.

[52] Clark M.E., He Z., Redding A.M., Joachimiak M.P., Keasling J.D., Zhou J.Z., Arkin A.P., Mukhopadhyay A., Fields M.W. (2012). Transcriptomic and proteomic analyses of *Desulfovibrio vulgaris* biofilms: Carbon and energy flow contribute to the distinct biofilm growth state. BMC Genom..

[53] Defoirdt T., Boon N., Sorgeloos P., Verstraete W., Bossier P. (2008). Quorum sensing and quorum quenching in *Vibrio harveyi*: Lessons learned from in vivo work. ISME J..

[54] Henke J.M., Bassler B.L. (2004). Three parallel quorum-sensing systems regulate gene expression in *Vibrio harveyi*. J. Bacteriol..

[55] Ng W.L., Perez L.J., Wei Y.Z., Kraml C., Semmelhack M.F., Bassler B.L. (2011). Signal production and detection specificity in *Vibrio* CqsA/CqsS quorum-sensing systems. Mol. Microbiol..

[56] Rezzonico F., Duffy B. (2008). Lack of genomic evidence of AI-2 receptors suggests a non-quorum sensing role for luxs in most bacteria. BMC Microbiol..

[57] Pereira C.S., Thompson J.A., Xavier K.B. (2013). Ai-2-mediated signalling in bacteria. FEMS Microbiol. Rev..

[58] Weiland-Bräuer N., Kisch M.J., Pinnow N., Liese A., Schmitz R.A. (2016). Highly effective inhibition of biofilm formation by the first metagenome-derived AI-2 quenching enzyme. Front. Microbiol..

[59] Surette M.G., Miller M.B., Bassler B.L. (1999). Quorum sensing in *Escherichia coli*, *Salmonella typhimurium*, and *Vibrio harveyi*: A new family of genes responsible for autoinducer production. Proc. Natl. Acad. Sci. USA.

[60] Aubert D.F., O′Grady E.P., Hamad M.A., Sokol P.A., Valvano M.A. (2013). The burkholderia cenocepacia sensor kinase hybrid AtsR is a global regulator modulating quorum-sensing signalling. Environ. Microbiol..

[61] Yarwood J.M., Schlievert P.M. (2003). Quorum sensing in *Staphylococcus* infections. J. Clin. Investig..

[62] Vuong C., Saenz H.L., Gotz F., Otto M. (2000). Impact of the agr quorum-sensing system on adherence to polystyrene in *Staphylococcus aureus*. J. Infect. Dis..

[63] Pratten J., Foster S.J., Chan P.F., Wilson M., Nair S.P. (2001). *Staphylococcus aureus* accessory regulators: Expression within biofilms and effect on adhesion. Microbes Infect..

[64] Nickzad A., Lepine F., Deziel E. (2015). Quorum sensing controls swarming motility of *Burkholderia glumae* through regulation of rhamnolipids. PLoS ONE.

[65] Blus-Kadosh I., Zilka A., Yerushalmi G., Banin E. (2013). The effect of pstS and phoB on quorum sensing and swarming motility in *Pseudomonas aeruginosa*. PLoS ONE.

[66] Krysciak D., Grote J., Orbegoso M.R., Utpatel C., Forstner K.U., Li L., Schmeisser C., Krishnan H.B., Streit W.R. (2014). RNA sequencing analysis of the broad-host-range strain sinorhizobium fredii NGR234 identifies a large set of genes linked to quorum sensing-dependent regulation in the background of a trai and ngri deletion mutant. Appl. Environ. Microbiol..

[67] Gao R., Krysciak D., Petersen K., Utpatel C., Knapp A., Schmeisser C., Daniel R., Voget S., Jaeger K.E., Streit W.R. (2015). Genome-wide RNA sequencing analysis of quorum sensing-controlled regulons in the plant-associated *Burkholderia glumae* PG1 strain. Appl. Environ. Microbiol..

[68] Sperandio V., Mellies J.L., Nguyen W., Shin S., Kaper J.B. (1999). Quorum sensing controls expression of the type III secretion gene transcription and protein secretion in enterohemorrhagic and enteropathogenic *Escherichia coli*. Proc. Natl. Acad. Sci. USA.

[69] Sha J., Pillai L., Fadl A.A., Galindo C.L., Erova T.E., Chopra A.K. (2005). The type III secretion system and cytotoxic enterotoxin alter the virulence of *Aeromonas hydrophila*. Infect. Immun..

[70] Schuster M., Lostroh C.P., Ogi T., Greenberg E.P. (2003). Identification, timing, and signal specificity of *Pseudomonas aeruginosa* quorum-controlled genes: A transcriptome analysis. J. Bacteriol..

[71] Decho A.W., Kawaguchi T., Chen Y.P. (2015). High Throughput *in vitro* Translation (Cell-Lysate Based) Assay for Detecting Quorum Sensing Signals. Patent.

[72] Kawaguchi T., Chen Y.P., Norman R.S., Decho A.W. (2008). Rapid screening of quorum-sensing signal N-acyl homoserine lactones by an in vitro cell-free assay. Appl. Environ. Microbiol..

[73] Montgomery K., Charlesworth J.C., LeBard R., Visscher P.T., Burns B.P. (2013). Quorum sensing in extreme environments. Life.

[74] Kimura N. (2014). Metagenomic approaches to understanding phylogenetic diversity in quorum sensing. Virulence.

[75] Brady S.F., Chao C.J., Clardy J. (2004). Long-chain N-acyltyrosine synthases from environmental DNA. Appl. Environ. Microbiol..

[76] Williamson L.L., Borlee B.R., Schloss P.D., Guan C.H., Allen H.K., Handelsman J. (2005). Intracellular screen to identify metagenomic clones that induce or inhibit a quorum-sensing biosensor. Appl. Environ. Microbiol..

[77] Kaksonen A.H., Spring S., Schumann P., Kroppenstedt R.M., Puhakka J.A. (2008). *Desulfotomaculum alcoholivorax* sp. nov., a moderately thermophilic, spore-forming, sulfate-reducer isolated from a fluidized-bed reactor treating acidic metal- and sulfate-containing wastewater. Int. J. Syst. Evol. Microbiol..

[78] Mayilraj S., Kaksonen A.H., Cord-Ruwisch R., Schumann P., Spröer C., Tindall B.J., Spring S. (2009). *Desulfonauticus autotrophicus* sp. nov., a novel thermophilic sulfate-reducing bacterium isolated from oil-production water and emended description of the genus *Desulfonauticus*. Extremophiles.

[79] LaRock C.N., Yu J., Horswill A.R., Parsek M.R., Minion F.C. (2013). Transcriptome analysis of acetyl-homoserine lactone-based quorum sensing regulation in *Yersinia pestis*. PLoS ONE.

[80] Khan R., Shen F., Khan K., Liu L.X., Wu H.H., Luo J.Q., Wan Y.H. (2016). Biofouling control in a membrane filtration system by a newly isolated novel quorum quenching bacterium, *Bacillus methylotrophicus* sp. WY. Rsc. Adv..

[81] Ivanova K., Fernandes M.M., Francesko A., Mendoza E., Guezguez J., Burnet M., Tzanov T. (2015). Quorum-quenching and matrix-degrading enzymes in multilayer coatings synergistically prevent bacterial biofilm formation on urinary catheters. ACS Appl. Mater. Interfaces.

[82] Garcia-Lara B., Saucedo-Mora M.A., Roldan-Sanchez J.A., Perez-Eretza B., Ramasamy M., Lee J., Coria-Jimenez R., Tapia M., Varela-Guerrero V., Garcia-Contreras R. (2015). Inhibition of quorum-sensing-dependent virulence factors and biofilm formation of clinical and environmental *Pseudomonas aeruginosa* strains by zno nanoparticles. Lett. Appl. Microbiol..

[83] Lee S., Park S.-K., Kwon H., Lee S.H., Lee K., Nahm C.H., Jo S.J., Oh H.-S., Park P.-K., Choo K.-H. (2016). Crossing the border between laboratory and field: Bacterial quorum quenching for anti-biofouling strategy in an MBR. Environ. Sci. Technol..

[84] Siddiqui M.F., Rzechowicz M., Harvey W., Zularisam A.W., Anthony G.F. (2015). Quorum sensing based membrane biofouling control for water treatment: A review. J. Water Proc. Eng..

[85] Chan K.-G., Liu Y.-C., Chang C.-Y. (2015). Inhibiting N-acyl-homoserine lactone synthesis and quenching *Pseudomonas* quinolone quorum sensing to attenuate virulence. Front. Microbiol..

[86] Shen G., Rajan R., Zhu J., Bell C.E., Pei D. (2006). Design and synthesis of substrate and intermediate analogue inhibitors of S-ribosylhomocysteinase. J. Med. Chem..

[87] Widmer K., Soni K., Hume M., Beier R., Jesudhasan P., Pillai S. (2007). Identification of poultry meat-derived fatty acids functioning as quorum sensing signal inhibitors to autoinducer-2 (AI-2). J. Food Sci..

[88] Hentzer M., Riedel K., Rasmussen T.B., Heydorn A., Andersen J.B., Parsek M.R., Rice S.A., Eberl L., Molin S., Hoiby N. (2002). Inhibition of quorum sensing in *Pseudomonas aeruginosa* biofilm bacteria by a halogenated furanone compound. Microbiol-Sgm.

[89] Katebian L., Gomez E., Skillman L., Li D., Ho G., Jiang S.C. (2016). Inhibiting quorum sensing pathways to mitigate seawater desalination ro membrane biofouling. Desalination.

[90] Almasoud A., Hettiarachchy N., Rayaprolu S., Babu D., Kwon Y.M., Mauromoustakos A. (2016). Inhibitory effects of lactic and malic organic acids on autoinducer type 2 (AI-2) quorum sensing of *Escherichia coli* O157:H7 and *Salmonella typhimurium*. LWT-Food Sci. Technol..

[91] Ibacache-Quiroga C., Ojeda J., Espinoza-Vergara G., Olivero P., Cuellar M., Dinamarca M. (2013). The hydrocarbon-degrading marine *Bacterium cobetia* sp. strain mm1ida2h-1 produces a biosurfactant that interferes with quorum sensing of fish pathogens by signal hijacking. Microbial Biotechnol..

[92] Abbas R. (2016). Personal communication.

[93] Galloway W.R., Hodgkinson J.T., Bowden S., Welch M., Spring D.R. (2012). Applications of small molecule activators and inhibitors of quorum sensing in gram-negative bacteria. Trends Microbiol..

[94] Teasdale M.E., Liu J., Wallace J., Akhlaghi F., Rowley D.C. (2009). Secondary metabolites produced by the marine bacterium *Halobacillus salinus* that inhibit quorum sensing-controlled phenotypes in gram-negative bacteria. Appl. Environ. Microbiol..

[95] Golberg K., Pavlov V., Marks R.S., Kushmaro A. (2013). Coral-associated bacteria, quorum sensing disrupters, and the regulation of biofouling. Biofouling.

[96] Liaqat I., Bachmann R.T., Edyvean R.G.J. (2014). Type 2 quorum sensing monitoring, inhibition and biofilm formation in marine microrganisms. Curr. Microbiol..

[97] Yang C.Y., Song G.J., Zhu Q., Liu S.J., Xia C.H. (2016). The influence of bacterial quorum-sensing inhibitors against the formation of the diatom-biofilm. Chem. Ecol..

[98] Santhakumari S., Kannappan A., Pandian S.K., Thajuddin N., Rajendran R.B., Ravi A.V. (2016). Inhibitory effect of marine cyanobacterial extract on biofilm formation and virulence factor production of bacterial pathogens causing vibriosis in aquaculture. J. Appl. Phycol..

[99] Mai T., Tintillier F., Lucasson A., Moriou C., Bonno E., Petek S., Magre K., Al Mourabit A., Saulnier D., Debitus C. (2015). Quorum sensing inhibitors from *Leucetta chagosensis* dendy, 1863. Lett. Appl. Microbiol..

[100] Nithya C., Pandian S.K. (2010). *Bacillus pumilus* of palk bay origin inhibits quorum-sensing-mediated virulence factors in gram-negative bacteria. Res. Microbiol..

[101] Chen R., Zhou Z., Cao Y., Bai Y., Yao B. (2010). High yield expression of an AHL-lactonase from *Bacillus* sp. B546 in pichia pastoris and its application to reduce aeromonas hydrophila mortality in aquaculture. Microbial Cell Factories.

[102] Seo M.J., Lee B.S., Pyun Y.R., Park H. (2011). Isolation and characterization of N-acylhomoserine lactonase from the thermophilic bacterium, *Geobacillus caldoxylosilyticus* ys-8. Biosci. Biotechnol. Biochem..

[103] Easwaran N., Karthikeyan S., Sridharan B., Gothandam K.M. (2015). Identification and analysis of the salt tolerant property of ahl lactonase (aiiatsawb) of *Bacillus* species. J. Basic Microbiol..

[104] Hiblot J., Gotthard G., Chabriere E., Elias M. (2012). Structural and enzymatic characterization of the lactonase sis lac from *Sulfolobus islandicus*. PLoS ONE.

[105] Merone L., Mandrich L., Rossi M., Manco G. (2005). A thermostable phosphotriesterase from the archaeon *Sulfolobus solfataricus*: Cloning, overexpression and properties. Extremophiles.

[106] Cao Y., He S., Zhou Z., Zhang M., Mao W., Zhang H., Yao B. (2012). Orally administered thermostable N-acyl homoserine lactonase from *Bacillus* sp. strain AI96 attenuates aeromonas hydrophila infection in zebrafish. Appl. Environ. Microbiol..

[107] Vinoj G., Vaseeharan B., Thomas S., Spiers A.J., Shanthi S. (2014). Quorum-quenching activity of the ahl-lactonase from *Bacillus licheniformis* dahb1 inhibits *Vibrio* biofilm formation in vitro and reduces shrimp intestinal colonisation and mortality. Marine Biotechnol..

[108] Mayer C., Romero M., Muras A., Otero A. (2015). Aii20j, a wide-spectrum thermostable N-acylhomoserine lactonase from the marine bacterium *Tenacibaculum* sp. 20j, can quench ahl-mediated acid resistance in *Escherichia coli*. Appl. Microbiol. Biotechnol..

[109] Morohoshi T., Tominaga Y., Someya N., Ikeda T. (2015). Characterization of a novel thermostable N-acylhomoserine lactonase from the thermophilic bacterium *Thermaerobacter marianensis*. J. Biosci. Bioeng..

[110] Huang W., Lin Y., Yi S., Liu P., Shen J., Shao Z., Liu Z. (2012). Qsdh, a novel ahl lactonase in the rnd-type inner membrane of marine *Pseudoalteromonas byunsanensis* strain 1a01261. PLoS ONE.

[111] Tang K., Su Y., Brackman G., Cui F., Zhang Y., Shi X., Coenye T., Zhang X.-H. (2015). Moml, a novel marine-derived N-acyl homoserine lactonase from *Muricauda olearia*. Appl. Environ. Microbiol..

[112] Tang K.H., Zhang Y.H., Yu M., Shi X.C., Coenye T., Bossier P., Zhang X.H. (2013). Evaluation of a new high-throughput method for identifying quorum quenching bacteria. Sci. Rep..

[113] Chow J.Y., Xue B., Lee K.H., Tung A., Wu L., Robinson R.C., Yew W.S. (2010). Directed evolution of a thermostable quorum-quenching lactonase from the amidohydrolase superfamily. J. Biol. Chem..

[114] Fetzner S. (2015). Quorum quenching enzymes. J. Biotechnol..

[115] Roy V., Fernandes R., Tsao C.Y., Bentley W.E. (2010). Cross species quorum quenching using a native AI-2 processing enzyme. ACS Chem. Biol..

[116] Maqbool T., Khan S.J., Waheed H., Lee C.H., Hashmi I., Iqbal H. (2015). Membrane biofouling retardation and improved sludge characteristics using quorum quenching bacteria in submerged membrane bioreactor. J. Membr. Sci..

[117] Oh H.S., Yeon K.M., Yang C.S., Kim S.R., Lee C.H., Park S.Y., Han J.Y., Lee J.K. (2012). Control of membrane biofouling in MBR for wastewater treatment by quorum quenching bacteria encapsulated in microporous membrane. Environ. Sci. Technol..

[118] Kalia V.C., Wood T.K., Kumar P. (2014). Evolution of resistance to quorum-sensing inhibitors. Microbial Ecol..

[119] Yeon K.M., Lee C.H., Kim J. (2009). Magnetic enzyme carrier for effective biofouling control in the membrane bioreactor based on enzymatic quorum quenching. Environ. Sci. Technol..

[120] Xu H., Liu Y. (2010). Control and cleaning of membrane biofouling by energy uncoupling and cellular communication. Environ. Sci. Technol..

